# What are the information needs of parents caring for a child with Glutaric aciduria type 1?

**DOI:** 10.1186/s12887-019-1742-x

**Published:** 2019-10-13

**Authors:** Hilary Piercy, Mildrid Yeo, Sufin Yap, Anthony R. Hart

**Affiliations:** 1 Sheffield Hallam University, Sheffield, UK; 2 Guys and St Thomas’ NHS Foundation Trust, London, UK; 3 Sheffield Children’s Hospital NHS Foundation Trust, Sheffield, UK

**Keywords:** Glutaric aciduria type 1, Metabolic condition, Parents, Information, Neurological outcome, Qualitative, Focus group

## Abstract

**Background:**

Newborn screening has enabled the early diagnosis of Glutaric aciduria type 1, with the possibility of improving neurological outcomes in affected children. Achieving those outcomes requires parents to effectively manage their child’s condition by adherence to a strict dietary regime and responding to situations that may trigger decompensation. The specific information and support needs of this group of parents are unknown.

**Methods:**

A focus group with five parents was conducted to gain insights into the information that parents needed and the ways in which they accessed and used information to manage their child’s condition. A topic guide was used to direct the discussion which was recorded and fully transcribed. All participants gave informed consent. Data were analysed using thematic analysis, a structured approach that contributes to transparency and validity of results while allowing the integration of predetermined and emerging themes. To ensure rigour, two researchers were involved in initial coding of data and key analytic decisions.

**Results:**

Two main themes were identified. ‘*Understanding the condition’* explored parent’s needs to understand the scientific complexity of the condition and to be aware of the worst case scenario associated with loss of metabolic control. ‘*Managing the condition’* explained how parents co-ordinated and controlled the involvement of other carers and parents’ need to be active partners in medical management to feel in control of the situation.

**Conclusions:**

The study highlights the importance of addressing parents’ initial and ongoing informational needs so they can fulfil their role and protect their child from metabolic harm.

## What is known?

- Early diagnosis of Glutaric aciduria type 1 (GA1) can improve neurological outcomes in affected children.
- Treatment involves adherence to a strict dietary regime and management of situations that can trigger decompensation.
- Achieving improved outcomes depends on parents.

## What is new?

- Parental perspectives on the difficulties of managing GA1.
- Insights into the types of information parents need to enable them to care effectively for their child and the challenges associated with accessing and comprehending that information.
- Insights into how clinicians could work with parents to help ensure improved neurological outcomes in children diagnosed with GA1.

## Background

Glutaric aciduria type 1 (GA1), is a rare metabolic condition with a prevalence of approximately 1 in 100,000 newborns. It is caused by a deficiency of the enzyme glutaryl-CoA dehydrogenase (GCDH), which is involved in the breakdown of the amino acids lysine, hydroxylysine and tryptophan. When there is little or no function of GCDH, a build-up of glutaric acid, 3-hydroxyglutaric acid, and glutaconic acid occurs, which are toxic to the brain.

GA1 traditionally presents with metabolic crisis and encephalopathy during infancy, leading to irreversible brain injury, resulting in permanent dystonia. Intelligence is usually spared initially till repeated injury. Early detection can potentially change the morbid natural history of this disease by enabling pre-symptomatic initiation of treatment and management regimes that reduce the likelihood of encephalopathic crises and improve neurological outcome [1–7] . Newborn screening for GA1 is established very widely around the world [8–10] and was incorporated into the Newborn Screening (NBS) programme in England and Wales in 2015.

Management of GA1 involves maintenance and emergency regimes. In the maintenance regime, dietary intake of lysine is strictly controlled and a supplementary amino acid (lysine free, low tryptophan) formula is given to provide sufficient protein for growth and development. Carnitine and micronutrients are supplemented [3]. Bloods are regularly monitored in outpatient clinics. In contrast, the emergency regime is instigated when a child is unwell, such as during infections, illness, immunisations or surgery, where there is a risk of encephalopathic crisis triggered by a catabolic state. The emergency regime involves timely admission to a metabolic ward for administration of high energy intake, reduction or omission of protein to prevent or reverse catabolic states, and additional carnitine supplementation. The early implementation of the emergency regimen and increased carnitine supplementation during intercurrent illnesses or other periods of metabolic stress remains the most important treatment in the prevention of neurological damage. Failure to adhere to these regimes is associated with poor neurological outcome [3].

Parents play a vital role in managing metabolic conditions to achieve the health outcomes that early detection through NBS offers. A limited body of evidence indicates the substantial associated burden. Gramer et al. (2014) surveyed parents living with a range of conditions detected through the NBS and found that dietary treatment and diagnoses with risk for metabolic decompensation despite treatment, were associated with higher perceived burden for the family [11]. The experience of raising a child with Medium chain acyl Co-A dehydrogenase deficiency (MCADD) and Phenylketonuria (PKU) identified challenges adhering to dietary regimes [12, 13]. In relation to GA1, parents are responsible for ensuring adherence to dietary treatment and in identifying and responding to situations that may trigger metabolic decompensation and poor neurological outcomes. Current international clinical guidelines for diagnosing and managing GA1 recognise that role and include recommendations for parental education and support including having ongoing direct access to metabolic specialists [14]. The informational needs of parents caring for a child with GA1 have not been explored. This article addresses that gap in knowledge. It arose from a public patient information (PPI) meeting with parents of children with GA1 and explored their informational needs in the context of discussions about proposed research.

## Methods

We used a descriptive qualitative study design [15], an approach that seeks to offer comprehensive summary of an event in the everyday terms of that event, to gain insights into the information that parents needed and how they had accessed and used information to manage their child’s condition. We recruited parents of children with GA1 from a regional metabolic centre and via a national parent’s metabolic support organisation. Parents were approached by their treating clinician or by an organiser of the support group and invited to participate. Given the small number of potential participants, this convenience sampling approach [16] was deemed most appropriate. Data collection involved a single focus group discussion. Its purpose, in terms of patient public participation, was explained prior to the discussion. We secured permission for recording and written informed consent from all participants.

Data collection involved a single focus group discussion, selected because we wanted to obtain rich data generated through group interaction. The discussion was facilitated by one of the project team (AH) with a topic guide to guide the discussion. Questions encouraged parents to reflect on their experiences from the point of diagnosis onwards in terms of what they had wanted to know about their child’s future, and to discuss where and how they had accessed information and the value of that information in enabling them to manage their child’s condition. The recorded discussion lasted 135 min, and was fully transcribed. Data were analysed using a thematic analysis approach [17]. This involved initial familiarisation, open inductive coding leading to development of a thematic structure with themes and sub themes. We all contributed to the analysis to ensure rigour. AH and MY performed the initial analysis. Preliminary themes were refined through an iterative process involving HP and SY.

Ethical approval was not required for the focus group because it was conducted for PPI purposes. However, in view of the rarity of the condition which limited anonymity, we registered the project as service development with a University Research Ethics Committee (ID: ER5861946, 14th March 2018). Written consent included agreement from all parents for publication of the findings.

## Results

### An overview of study participants

Five parents (4 mothers and 1 father) of 4 children contributed to the discussion. Their children (pseudonymised) were: Rachel, 17 years, Alex, 8 years, Peter, 3 years, Matthew 2 years.

### Themes

Two themes were identified: *‘understanding the condition’* and *‘managing the condition.’*

### Understanding the condition

Parents needed to understand the condition sufficiently to be able to manage it on a day to day basis and to protect their child from metabolic harm. This involved ‘grappling with the science’ and being aware of the ‘worst case scenario.’

### Grappling with the science

The parents explained how, when presented with a diagnosis of GA1, they needed to understand what the condition was about. The rarity and the scientific complexity of the condition made this very challenging. Readily available internet sources provided an overview of the condition but were inadequate for their needs. As Peter’s mother explained, *“we wanted concrete evidence and we wanted direct research papers*.” These scientific papers were hard to access and extremely difficult for the parents to understand. It had taken Rachel’s mother *“not being a biochemist, quite a long time to read through it and work it out.”* She justified the importance of grappling with this level of scientific information, despite the evident challenges:

*Whether you understood it or not was something else but at least we had something to go on … solid hard scientific stuff … You need to know what you're doing and what you're trying to do …*

Parents also identified the importance of having that scientific information translated into practically focused, written information that would help them manage the condition on a daily basis. A booklet written by a physician from the USA provided one family with guidance for *‘sick days management ‘*, and had been an invaluable reference source for Rachel’s mother:

*If I didn’t know what to do, I'd just go back and look at it … . Sometimes, when you're not sure, it's just clear, 'is it one of those things that might cause a problem', ‘do I need to worry?’ So yes, I had all the science, but I also had this handy little guide.*

### Worst case scenario

Managing a child with GA1 involves being alert to the ever present threat of metabolic crisis, which can lead to irreversible neurological damage. Parents explained that these worst case scenarios dominated most online information sources. Although they had found this *‘terrifying’* they emphasised how important it was for parents to grasp the enormity of the situation so they could understand the importance of their role in managing the condition and preventing that worst case scenario. As Peter’s mother stated, *“It’s the end of your life, the end of your chance of a normal life, and they need to know that.”*

The parents whose children had been diagnosed clinically, expressed concerns about how NBS might influence parental perceptions of the condition and reflected on the possible implications:

*My son had a crisis. We had no idea he had the thing so no-one is responsible, but if I knew he had this condition and he subsequently had a crisis because I hadn't acted fast enough or perhaps thought that things were OK, then I'd never be able to forgive myself. You need to know. (Peter's mother)*

### Managing the condition

Parents managed the condition on a daily basis by ensuring that the strict dietary regime was adhered to. This involved ‘co-ordinating and controlling intake and involvement’. However, they needed to be ‘active partners in medical management’ to feel sufficiently in control of the situation.

#### Co-ordinating and controlling intake and involvement

Within the family home, activities were carefully co-ordinated to ensure adherence to the strict dietary regime. Peter’s family used a checklist on the fridge and Matthew’s family used a whiteboard to record actions and prevent omissions or duplications:

*We’ve got a big whiteboard in the kitchen and everything goes up there. So, we’re not confused between each other. If I’ve done Levo [levo-carnitine - a dietary supplementary] I put it up there, if [another family member] has done the jab [injection of Levo-carnitine], she puts it up there. So, we can go to that and see that he’s had such and such or that he needs … (Matthew's mother)*

As the child grew older, there was a need to involve others in caring for their child. The extent to which other family members were involved was limited, either because the responsibility was too great for them or because they could not be trusted to adhere to the dietary regime:

*They [the grandparents] did not want that responsibility because they were terrified that if anything happened, relationships would be destroyed. It’s a huge responsibility. (Rachel's mother)*

*That’s been sometimes quite a bit of an issue when we go to family - trying to give them a little bit meat or something else – saying it won’t hurt! (Alex's mother)*

At some point, however, parents had to delegate some degree of responsibility for managing the condition to carers and teachers. This involved making decisions about who they felt they could trust, ensuring they provided those carers with information in written and verbal formats to enable them to manage the condition, and then working with them to develop confidence and build trust relationships:

*The main things that we said … if he’s not himself you would have to call us immediately, or if you spot a temperature please medicate him immediately and then there’s the diet – do not feed him any foods that we haven’t provided. (Matthew's father)*

*I went in to the school and met them as well to get it over to them how important it was. I said I’d rather them call me a million times. (Alex's mother)*

### Active partners in the medical management

As partners in care, parents and clinicians needed to develop a shared understanding of medical management and a common language to discuss it. A fundamental part of this process was achieving a shared understanding of metabolic stability which was the primary management goal:

*Also, something which took me a long time to pick up on, when she was young, was this thing about was she ill? Now what I thought was ill and what the Metabolic Team meant by ill were two different things … .when they asked me if she was ill – they meant was she metabolically unwell. (Rachel's mother)*

Ongoing management of GA1 involves making regular changes to the dietary regime in response to the results of regular blood tests. Alex’s mother described the process:

*Alex probably has hers every 3 months and then we generally get a phone call if we need to change her diet or if her number of protein exchanges is OK.*

All the parents had some concerns about this approach and wanted all the test results to be shared with them regularly. They suggested this would serve two main purposes. First, it would give them some sense of how their child was progressing, and secondly the act of receiving those results would provide them with reassurance that the results had been processed and interpreted.

*Just to perhaps see a pattern may be for the parents to see why they were high and look back on what they've had to eat that day or that week. (Alex's mother)*

*You hope that they, whoever’s dealing with your child, would pick up any anomalies in the results. But I’m not 100% confident that that would always happen, as mistakes are made. So, I’d like that information. (Peter's mother)*

### Strengths and limitations

The rarity of the condition made recruitment challenging and necessitated the use of convenience sampling. Using two approaches to recruitment enabled us to conduct one focus group. Within this constraint participants included parents with older children who had been diagnosed clinically and parents with children diagnosed through NBS which contributed to richness of the data.

Notwithstanding this, the scale of the study, sampling approach and method of data collection are study limitations which restrict generalisability of findings and claims of saturation. An enhanced recruitment strategy to identify and recruit a larger number of participants and either conducting more focus group discussions or individual semi-structured interviews would have provided a broader range of experiences and viewpoints and generated richer data. Additionally, a purposive sampling approach and collecting demographic information, including educational background to ensure we recruited participants from a wider demographic background, would have enabled us to achieve data saturation.

## Discussion

The findings offer insights into the substantial informational challenges experienced by parents raising a child with GA1. Firstly, those associated with achieving a detailed understanding of the condition itself as well as the management regimes and secondly those associated with effective information sharing within family and social relationships in order to protect their child from the potential consequences of poor management.

The improved outcomes that newborn screening offers for those families affected by GA1 depends on adherence to the dietary regime and rapid responses to metabolic imbalance [3, 14] Several studies involving Phenylktonuria (PKU), a metabolic condition comparable to GA1 insofar as it is managed by restricting dietary intake of specific amino acids, have demonstrated a positive association between parental knowledge of the condition and metabolic control, an indicator of adherence to the dietary regime [18–20]. The implications of inadequate parental knowledge are more profound for GA1 because this condition has a more acute clinical course than PKU, if treatment is sub-optimal. The parents in this study wanted highly complex information in the post diagnostic period, which was more detailed and in-depth than that they had received from their metabolic team. This level of information enabled them to achieve the sense of control they needed to manage the condition and keep their child safe. Other studies have similarly identified discrepancies in the amount of in-depth information parents need and receive from health providers in the post diagnostic period [21, 22]. Although parents commonly use internet information sources to supplement information received from healthcare providers, to reduce their anxiety and to enable them to cope better with their child’s condition [23], they prefer to receive information about their child’s condition from a trusted health care provider rather than the internet [24]. Studies indicate that parents want information in a variety of formats [22] which enables them to cope better with their child’s condition. A randomised control study involving parents of children with food allergies found that those receiving a food allergy handbook for parents had greater levels of knowledge and confidence, and better quality of life than those in the control group [25].

The level of understanding acquired by the parents in our study is unlikely to be accessible to all parents in their situation. A study involving 42 children with PKU, reported significantly higher phenylalanine levels (indicating poor adherence to dietary regime) if mothers had lower levels of education [26]. Our findings indicate the importance of written materials for parents that contain sufficient amounts of scientific information translated into an accessible format and clearly linked to information about how to manage the condition. Some materials are now freely available for parents that address this need [27, 28]. To ensure that they are fit for purpose, all materials should be developed using a co-production approach and involve parents of children with GA1, the charities established to support them, as well as members of the multidisciplinary clinical team including physicians, dieticians and specialist nurses.

The parents also wanted to be actively involved in monitoring their child’s metabolic status and to receive details of the test results on which dietary management decisions were based. They wanted to share in the medical management and were anxious because they did not receive test results, fearing that abnormal results may have been missed by clinicians. Other studies have highlighted the anxieties associated with receiving them. This relates to the twofold role that test results play in managing metabolic conditions. As well as informing dietary changes, they indicate how well the current diet has been adhered to, effectively providing feedback on parental performance. This feedback element is associated with considerable anxiety for parents of children with PKU, particularly when faced with a child that refuses to eat [13]. These findings suggest the need for a more detailed insight into how parents of children with GA1 understand and want to engage with test results which could then help to inform ongoing service improvements.

The extent to which friends and family were involved in care was very limited. When they needed to involve others in care, parents assumed educational responsibilities towards the carers. Similar findings have been reported for other conditions with high treatment management responsibilities, with parents issuing detailed instructions, preparing educational packs, and spending considerable amounts of time with nursery and school staff to help them understand the key elements of the condition [12, 13, 29]. The responsibility for this is substantial and likely to be daunting for some. Support from specialist nurses was highly valued by parents of child with MCADD because they were able to able to convey information in a balanced and non-emotive way [12]. This type of support may also benefit parents of children with GA1.

## Conclusion

Early detection of GA1 through the NBS programme enables good health outcomes but this is dependent on strict adherence to dietary regimes, especially when unwell. Parents have a pivotal role in that process. Insights into their informational needs are valuable for clinicians to inform the ongoing care and support they provide for parents to enable them to fulfil their role and protect their child from metabolic harm. The complexity of the scientific information makes this particularly challenging highlighting the importance of co-production approaches for any initiatives to develop materials or ways of working.

## Data Availability

The datasets generated and/or analysed during the current study are not publicly available due to the nature of the data and anonymity considerations but are available from the corresponding author on reasonable request.

## Abbreviations

GA1: Glutaric aciduria type 1
GCDH: Glutaryl-CoA dehydrogenase
MCADD: Medium chain acyl Co-A dehydrogenase deficiency
NBS: Newborn Screening
PKU: Phenylketonuria
PPI: Patient public information
